# Remote sensing and conservation of isolated indigenous villages in Amazonia

**DOI:** 10.1098/rsos.140246

**Published:** 2014-11-05

**Authors:** Robert S. Walker, Marcus J. Hamilton, Aaron A. Groth

**Affiliations:** 1 Department of Anthropology, University of Missouri, Columbia 65211, MO; 2 Santa Fe Institute, Santa Fe, NM 87501, USA; 3 Department of Geography and the Environment, University of Texas at Austin, Austin, TX 78712, USA

**Keywords:** uncontacted indigenous societies, land use, satellite imagery, Amazonia

## Abstract

The vast forests on the border between Brazil and Peru harbour a number of indigenous groups that have limited contact with the outside world. Accurate estimates of population sizes and village areas are essential to begin assessing the immediate conservation needs of such isolated groups. In contrast to overflights and encounters on the ground, remote sensing with satellite imagery offers a safe, inexpensive, non-invasive and systematic approach to provide demographic and land-use information for isolated peoples. Satellite imagery can also be used to understand the growth of isolated villages over time. There are five isolated villages in the headwaters of the Envira River confirmed by overflights that are visible with recent satellite imagery further confirming their locations and allowing measurement of their cleared gardens, village areas and thatch roofed houses. These isolated villages appear to have population densities that are an order of magnitude higher than averages for other Brazilian indigenous villages. Here, we report on initial results of a remote surveillance programme designed to monitor movements and assess the demographic health of isolated peoples as a means to better mitigate against external threats to their long-term survival.

## 2. Introduction

Brazil’s indigenous agency, Fundação Nacional do Índio (FUNAI), periodically releases information concerning locations of isolated peoples, also known as ‘uncontacted Indians’ or ‘indigenous people in voluntary isolation’ [1,2]. The long-term survival of isolated peoples hangs in a delicate balance. Previous contacts with encroaching Europeans have all been devastating for indigenous populations [3–6]. Disease, displacement, deforestation, mining, narcotrafficking and hydrocarbon extraction are currently some of the major threats to indigenous societies [1–12]. Limited information is available as to the demographic health of populations that remain isolated, but most are likely to be critically endangered and facing many of the same external threats that have plagued indigenous populations in the region for the past 500 years [1,2].

There is a concentration of isolated indigenous groups on the Brazil/Peru border in the headwaters of the Envira River that includes the Mashco-Piro nomadic hunter–gatherers and a number of Pano-speaking horticultural societies [1,7]. Most of the information for these Pano speakers comes from overflight surveillance of their villages and gardens which began as early as 1998 by FUNAI [1]. Photographs and videos of villagers shooting bows-and-arrows at planes or fleeing for cover have proven important for documenting their locations and gaining international interest in their conservation. It has been surmised that some isolated people in this region have been recently forced to flee into the state of Acre, Brazil because of invading drug and timber smugglers in Peru who have pushed into indigenous lands [7–12]. Many watersheds have been impacted by logging in both Peruvian protected areas and territorial reserves that have been set aside for isolated indigenous populations. Logging is not permitted in protected areas, but loggers continue to operate with impunity inside park boundaries [8–10], and logging activities are purportedly financed by narcotrafficking [10–12].

Population estimates are crucial information for assessing trends in the demographic health of isolated indigenous populations, but for obvious reasons there is considerable difficulty in obtaining accurate counts. Overflights are one strategy, but entail high costs and are invasive given that they instil reactions of fear in isolated people. Fortunately, remote sensing overcomes these issues. The methodology for estimating population size from spatial data (i.e. field areas, number of houses or room area) is a well-developed approach in anthropology [13–19]. Here, we demonstrate the utility of satellite imagery to locate isolated villages, estimate their local village-level population densities and track the growth of one particular village over time. These are some of the last truly indigenous economies of the world, disconnected from outside market influences, and therefore their spatial ecology is the best available analogue for pre-Columbian land use.

## 3. Methods

Locations of isolated indigenous villages were pieced together from FUNAI reports and news stories stemming from overflights. Landsat maps of recent deforestation [20] and other satellite images with high resolution (e.g. TerraServer) were scoured for forest clearings. At least five isolated communities were located in the border region between Brazil and Peru in the headwaters of the Envira River that correspond to confirmed locations released by FUNAI and that contain thatched roof houses. This region is the most monitored by FUNAI and hence population estimates are available from their frequent overflights. While there are more isolated villages we have found with satellite imagery, population estimates are unavailable to us. (Given the sensitive nature of this information, we do not reveal locations of isolated villages; we are cautious, because adventurers, missionaries, narcotraffickers, loggers and settlers have been known to contact isolated people and cause them harm.)

We purchased 50 cm resolution WorldView or GeoEye satellite images from DigitalGlobe for each of the five sites. The overall cleared areas including swidden (slash and burn) horticultural fields, and village areas were measured with the polygon measurement tool in ArcGIS v. 10.2.1. If vegetation within or surrounding cleared areas was clearly composed of planted crops as opposed to naturally occurring forest, we included these areas in our estimates. We used the image analyst tool in ArcMap to produce better images and measurements of the houses.

The isolated village areas are compared against a sample of 71 other indigenous Brazilian villages (7527 total residents) where it was possible to match locations with population estimates [15]. These villages are all in forested regions making cleared areas easy to demarcate. For reasons of sufficient resolution of satellite imagery in Google Earth and well-documented village locations, the most sampled regions are Northwest Brazil, Xingu River Basin and Yanomami land in Northern Brazil. For a subsample of 25 villages, satellite imagery is of high enough resolution to allow measurement of the area of houses (and other buildings) and hence estimates of the house area per person.

## 4. Results

**Figure 1.**
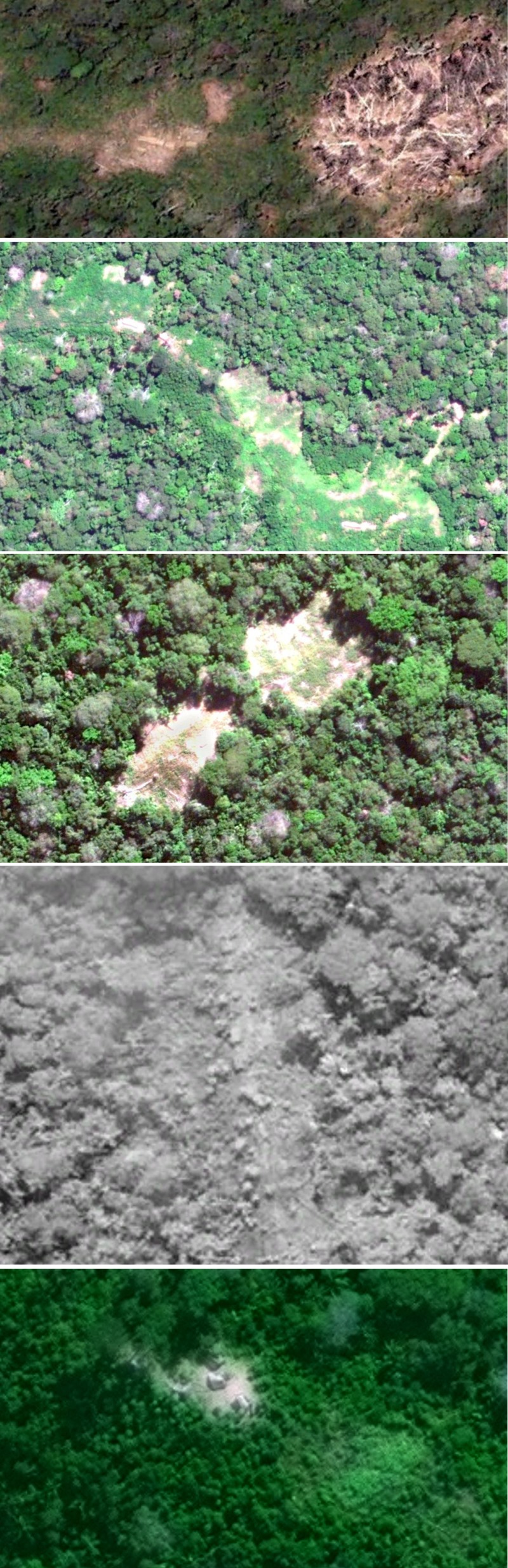
Satellite images of clearings and houses for sections of the five sites. From top to bottom are sites X, H, M, F1 and F2. © 2014 DigitalGlobe, Inc.

**Figure 2.**
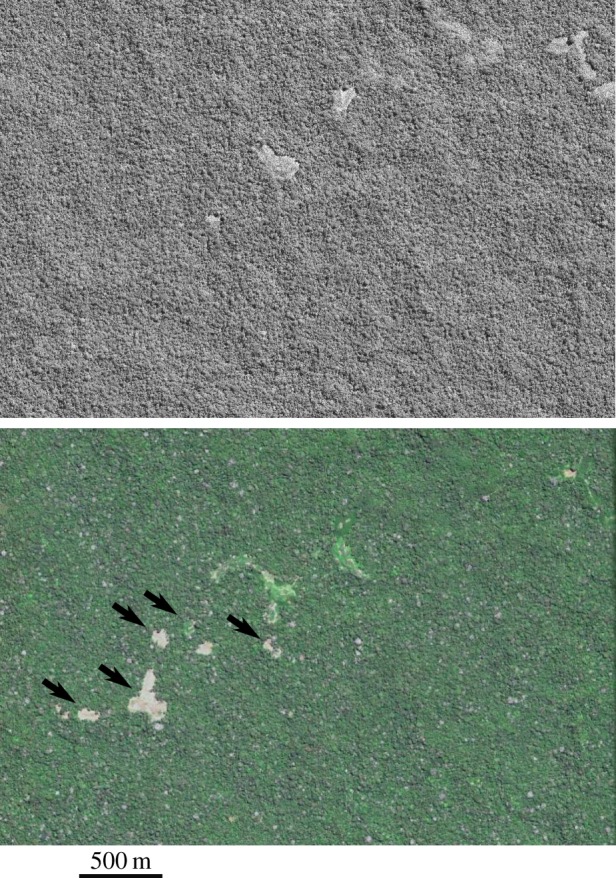
Top image is the western portion of site H in May 2012 and the bottom image is the same area in July 2013. Note the addition of more slash-and-burn fields (designated by arrows) and the expansion of the two large cleared areas in the centre of the image. These additions total 16 ha cleared in 14 months. Areas that were cleared in 2012 are filled with planted crops in 2013. © 2014 DigitalGlobe, Inc.

The five isolated indigenous villages are given names according to nearby water sources, but abbreviated here to maintain secrecy (table 1 and figure 1). These swidden horticultural communities range from small villages of approximately 50 people to a large and growing village of approximately 300 people. (i) Site X is a small community of Yaminawa (‘Xatanawa’ dialect) that originated in Peru and fled several massacres. The village is visible in 2006 satellite imagery, but was abandoned and overgrown in 2012 imagery [15]. An overflight confirmed them to be at a nearby location in March 2014. Recent reports that surfaced after contacts with an Asháninka village starting in June 2014 indicate that there are around 60 people from this village. (ii) Site H is a large village that according to FUNAI has doubled in population over the past 20 years. It is approximately 30 ha in total cleared area and houses an estimated 300 people. Satellite images from May 2012 and July 2013 show that the western portion of site H added an additional 16 ha of cleared area, increasing in size from 12 to 28 ha in 14 months (figure 2). The eastern portion of the village, not shown, mostly grew fallow over the same period. (iii) Site M represents at least five separate locations spread over approximately 18 km and appears to be part of the same extended group as site H given that several smaller clearings reside in between these two locations. (iv) Site F1 is the one location in Peru. This site is important to document, because our previous understanding was that most sedentary (horticultural) isolated people had fled into Brazil. The image we measured is from 2012, but they are in the same location as verified by an overflight in August 2014. (v) Site F2 is arguably the most isolated group in Acre with limited available information other than an estimate of around 50 people. Reportedly, there are other villages nearby that we were unable to locate. Pictures from a FUNAI overflight clearly shows a village that differs in structure from the one we located.

**Table 1.** Information for five different locations of isolated indigenous villages. Population estimates are based on FUNAI accounts.

site	language/dialect	confirmed in overflight	satellite image date	*n* clearings	*n* houses	cleared area (ha)	population estimate
X	Xatanawa	2008, 3/2014	5/2006	3	4+	3.8	∼60
H	Yaminawa?	2008, 7/2009	7/2013	11	10+	30.9	∼300
M	Yaminawa?	2008	7/2013	20	?	17.7	∼100
F1	Mastanahua?	8/2014	8/2012	3	9?	1.8	?
F2	Mastanahua?	2008, 7/2009	7/2013	4	5	3.3	∼50
total				41	28+	57.7	510

The 4+ houses in site X (there are multiple huts in a line), and the five houses in site F2 have total house areas of 112 and 128 m^2^, respectively (the other sites have too many houses to locate and measure accurately). Assuming that FUNAI estimates of 50–60 people in each community are accurate, there is about 2 m^2^ of living space per individual within a house. In comparison, the sample of 25 other Brazilian communities with house measurements yields a mean house space that is considerably larger at 17 m^2^ per person, at least, in part because these villages contain non-residential buildings such as sheds, churches, schools and health clinics.

**Figure 3.**
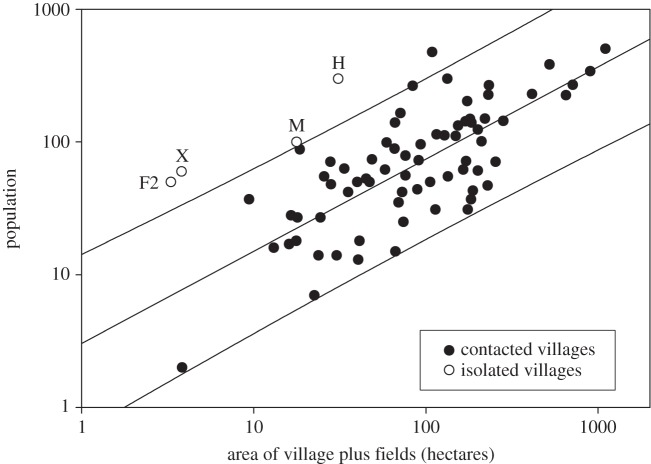
Population-by-area graph for 71 (contacted) indigenous villages in Brazil with known populations and areas measured using high-resolution images in Google Earth. Best-fitted regression line (*y*=3.04 *x*^0.694^, *R*^2^=0.55) and 95% prediction interval are shown. Isolated villages are marked separately with site letters using areas and population estimates from table 1. Note the log–log scale and that isolated villagers live at a high density, all appearing above the upper 95% prediction interval.

The population-by-area measurements for a sample of 71 (contacted) indigenous villages in Brazil shows a log–log relationship (figure 3). The isolated villages all fall above the 95% prediction interval indicating that these are extremely high density villages. Overall, the 71 villages have a *per capita* area usage of 1.44 ha, whereas the isolated villagers are using only an estimated 0.11 ha each. Hence, population densities are approximately an order of magnitude greater in isolated villages than in contacted villages. One explanation for this result might be that FUNAI has systematically overestimated the population sizes of the isolated villages. However, it is unlikely that FUNAI has overestimated population sizes by an order of magnitude. Further, we find the FUNAI population estimates to be credible given the number and size of the houses visible in satellite and overflight images.

## 5. Discussion

We show that satellite imagery can contribute to the study of isolated indigenous peoples by providing evidence for their locations and measurements of their villages, gardens and houses. Isolated villagers appear to live at high densities in people per cleared area (approx. 9 people ha^−1^), over an order of magnitude higher than the average for other Brazilian indigenous villages (approx. 0.7 people ha^−1^). Likewise, the house area per person is only 2 m^2^ in the isolated villages but averages 17 m^2^ per person for other Brazilian indigenous communities. These differences are probably due to a combination of factors including the difficulties associated with clearing trees using traditional technologies, the degree of traditional housing and subsistence, and the age of communities. Traditionally, tree cutting was done with stone axes which is extremely time consuming [21–24]. FUNAI gave steel machetes and axes to isolated populations for decades (this practice has since been discontinued in this region), and they have also been stolen from settlers and other indigenous communities. Machetes and metal pots are visible in some photographs taken during overflights of isolated villages. Other Brazilian communities have better access to steel tools, as well as chainsaws and tractors in some places, greatly easing the time cost of clearing villages and gardens, and they have better access to long-lasting building materials. Moreover, the isolated villages are probably young in age (a few years to a decade, with the exception of site H which is several decades old), whereas most of the other Brazilian villages we measured have often been in the same place for longer periods of time (several decades or longer). Older and more acculturated communities develop permanent amenities such as soccer fields, airstrips and non-residential buildings for schools, churches and health clinics, and they clear more trees for firewood. It is possible that the high population density of isolated villages is an indicator of their precarious situation where they are constantly worried about self-defence and are unwilling to spread out for fear of attacks. Alternatively, their subsistence may simply rely more on traditional hunting and gathering and less on horticulture, and lacks the cash cropping that is done in other villages. A reliance on more fishing is unlikely to be a factor given that these isolated villages are not on large rivers. Regardless, knowledge of the high population densities is important for estimating populations in isolated villages directly from satellite images in places where overflights have not been conducted. Moreover, they may indicate a need to increase estimates of pre-Columbian populations that are based, at least in part, on densities of contemporary indigenous populations [4].

Overflights of isolated villages entail a number of costs. First, aircraft run the risk of mechanical failure and flights are expensive (currently approx. $1500 h^−1^). Second, overflights are invasive often causing villagers to either flee in fear or to shoot bows-and-arrows at the plane. Finally, while colour photographs of villagers provide wonderful details and attract valuable media coverage, overflights often lack a systematic data collection methodology. Remote sensing overcomes all of these issues given that it is inexpensive (50 cm images cost approx. $10 km^−2^), entails no risk, and is completely non-invasive. Furthermore, satellite images facilitate systematic data collection as we have done here by measuring the cleared areas of villages and gardens and the sizes of houses. The major benefit remains, namely incontrovertible evidence of the time-stamped locations of isolated peoples.

One difficulty with remote sensing is that windfalls, landslides and invaders also create forest clearings. For example, other clearings on the Peru–Brazil border in areas with confirmed FUNAI reports of isolated people have no clearly visible human-made structures and are not included here. There are another 30+ clearings in the hilly region in Peru’s Alto Purús National Park visible in Google Earth that a recent overflight found to be all natural with no evidence of human settlement. High-resolution imagery generally reveals evidence of human structures if they are present, which in this region are the classic Pano-style rectangular longhouses or a series of houses in a cluster or line. While drug runners and loggers also construct houses, their houses are less communal and lack the horticultural fields that surround indigenous villages. Another difficulty with remote sensing is that satellite images cannot easily detect nomadic hunter–gatherers such as the Mashco-Piro. Abundant evidence has emerged for these nomads not from satellite images or overflights but from chance encounters and temporary huts along rivers in both Peru and Brazil. For example, over 100 Mashco-Piro camped near the community of Monte Salvado, Peru in 2013. However, the vast majority of indigenous groups in Amazonia, including those that are still isolated such as the Pano speakers studied here, are slash-and-burn horticulturalists and therefore detectable with remote sensing.

Small, isolated villages such as those at sites F1, F2 and X face an imminent threat of falling below a minimum viable population. It is heartening to know that there are multiple villages in this region, but it is not clear if they are friendly to one another or likely to exchange marriage partners. While the large size and recent growth of site H may at first appear to be a positive indicator of demographic health, FUNAI’s interpretation is that this is a result of immigrants fleeing from other areas. External pressures are encroaching in several directions, including the invading drug and timber smugglers coming from Peru and pressure by loggers and colonists from the new road that connects Seringal Novo Porto to the town of Jordão in Acre, Brazil only 30 km as the crow flies from site H.

Most if not all of the other some 50–100 isolated indigenous peoples in Greater Amazonia face dire situations in terms of small populations struggling against an onslaught of external risks [1,2]. All surviving Brazilian indigenous populations suffered extensive mortality during contact with the outside world [4–6]. Fortunately, satellite imagery is obtainable for many more isolated populations, and as more satellite imagery becomes available we plan to track their area usage over multiple years as we have done with site H. Other remote sensing techniques such as infrared, LIDAR, aerial surveillance or facial recognition may also prove useful in the near future provided ethical concerns are adequately addressed. By providing real data on the relationship between external threats (i.e. deforestation, illegal mining, cattle ranching, narcotrafficking, etc.) and indigenous population dynamics and spatial ecology in the upcoming years, we can use remote sensing information in strategic ways to provide tools for improved governmental policies. Indigenous land rights and security are key; it is crucial to set aside and enforce appropriately sized protected areas. Our long-term goal is a longitudinal surveillance programme across Amazonia that can facilitate informed decisions by policy-makers to increase protection of isolated populations.
